# Producing Mobility, Withholding Authority: Epistemic Drain and Nursing Sovereignty in Nepal

**DOI:** 10.1111/nup.70093

**Published:** 2026-06-16

**Authors:** Animesh Ghimire

**Affiliations:** ^1^ School of Nursing and Midwifery, Faculty of Medicine, Nursing and Health Sciences Monash University Clayton Australia; ^2^ School of Nursing and School of Public Health Chitwan Medical College Chitwan Nepal; ^3^ Sustainable Environment Foundation Nepal (SEFNep) Kathmandu Nepal

**Keywords:** brain drain, education, emigration and immigration, Gen AI, Nepal, nursing, philosophy, professional autonomy

## Abstract

Global discussions of nursing in low‐ and middle‐income countries (LMICs) are dominated by the language of shortage, staffing, retention, and migration. Although useful, this technocratic vocabulary obscures a deeper philosophical crisis: nurses may be numerically produced yet institutionally denied authority over knowledge, care, and professional futures. Using Nepal as a theory‐generating case, this conceptual article employs critical interpretive synthesis and philosophical analysis of nursing, policy, migration, and interdisciplinary scholarship to argue that the central problem is not workforce scarcity alone but a crisis of ‘nursing sovereignty’. The paper introduces three concepts. First, epistemic drain extends the idea of brain drain by naming the erosion of mentorship, pedagogical continuity, professional memory, policy voice, research capacity, and the public credibility of nursing knowledge. Second, professionalised for exit captures a paradox of production in which nursing education expands global mobility more readily than local authority, making migration a morally justified reaction to constrained socioeconomic prospects, rather than a lack of professional dedication. Third, nursing sovereignty names the collective capacity of nurses to shape the ends, authority, knowledge, and institutional conditions of their profession. Drawing together social epistemology, feminist ethics, decolonial lens, political economy, sociology of professions, spatial theory, and digital pedagogy, the article argues that Nepal illuminates a wider LMIC condition in which nurses are essential yet disposable, mobile yet institutionally voiceless, and professionalised yet epistemically diminished. It concludes with a six‐domain normative framework for institutional redesign: epistemic authority, educational integrity, governance voice, material justice, spatial and digital parity, and mobility justice. Reframing nursing in this way shifts debate from retention to justice, from labour supply to institutional re‐design, and from workforce management to the political philosophy underpinning nursing practice.

## 1 Introduction: From Shortage to Sovereignty

Nursing in LMICs is often discussed with a focus on quantitative metrics but philosophically penurious terms: shortage, staffing, retention, and migration. That vocabulary effectively conveys the extent of workforce shortages, yet it fails to address the underlying structural dynamics at play. While it quantifies the nursing deficit, it neglects to analyse the institutional frameworks that render these professionals disposable, interchangeable, or subject to international recruitment. Existing global data indicate that simply calculating workforce numbers is inadequate in capturing the complexities of this issue. Nurses remain the largest occupational group in the health workforce; the global nursing workforce grew from 27.9 million in 2018 to 29.8 million in 2023, but this growth has not dissolved inequality. Currently, 78% of the global nursing workforce is concentrated in countries that account for just 49% of the world's population. This disparity highlights the ongoing challenges faced by LMICs in their efforts to educate, employ, support, and retain nursing professionals within their health systems under equitable conditions (WHO 2025b, 2025d).

The dominant literature on nurse migration from lower‐resource settings is indispensable, but it often remains bounded by a technocratic logic. Systematic reviews show that nurse migration from LMICs is strongly shaped by poor remuneration, insecure and under‐resourced workplaces, limited career prospects, job dissatisfaction, and broader political and security conditions (Konlan et al. 2023; Toyin‐Thomas et al. 2023). Meanwhile, integrative and qualitative analyses of nursing policy engagement in LMICs reveal a persistent underrepresentation of nurses in policy development; instead, their roles are predominantly relegated to policy implementation processes (J. Etowa et al. 2023; Rasheed et al. 2020). Yet a more balanced reading of the field also matters. Migration is not interpreted everywhere as pure loss. Some scholarship points to remittances, diaspora linkages, and possible forms of skills circulation as partial benefits for migrant households and, in some cases, for source countries (Lorenzo et al. 2007; Squires and Amico 2014). Agency‐based migration theory likewise cautions against treating movement as an issue in itself, emphasising the importance of aspirations and capabilities that shape the freedom to move or to stay (de Haas 2021). Broader scholarship on health systems strengthening makes a related point: workforce crises cannot be reduced to emigration alone because they are also problems of governance, financing, information, absorptive capacity, and institutional design (Adam et al. 2012; WHO 2025c). This article does not reject those perspectives; rather, it argues that they remain incomplete unless they are brought into conversation with questions of authority, recognition, and the political conditions under which nursing knowledge is permitted to count.

Recent nursing philosophy and interdisciplinary scholarship sharpen this question by shifting attention from labour supply alone to hierarchy, epistemic legitimacy, and institutional design. Dunn (2025) argues that nursing's persistent subordination is not a historical residue but the effect of institutionalised power/knowledge asymmetries embedded in architecture, governance, credentialing, and professional hierarchy. Jackson (2026) extends this insight to nursing academia, showing how managerial control and audit cultures reproduce class‐ and gendered hierarchies while eroding intellectual freedom. Rosario and colleagues caution that the term “decolonisation” diminishes in significance when it is merely equated with diversity or inclusion. They argue that it should instead be understood as a critical examination of colonial power dynamics, epistemic hegemony, and the necessity for material redistribution (Rosario et al. 2024). Additionally, Woodly et al. (2021) argue that care should not be viewed solely as a private virtue; rather, it is a political and material practice that plays a crucial role in shaping collective existence. The consequence of reading these literatures together is a shift in the problem definition itself: the crisis of nursing in LMICs is not only a matter of labour supply but also of professional authority, epistemic legitimacy, and institutional design.

Nepal prominently highlights this paradox, as of 24 February 2026, the Nepal Nursing Council (NNC) reported 126,889 registrations across nursing and midwifery categories, including 86,358 Registered Nurses (NNC 2026). This figure initially suggests a surplus of qualified practitioners. Yet the WHO human resources for health profile for Nepal identifies international migration as a “significant challenge” while also highlighting uneven distribution, high mobility, insufficient and vacant public posts, and limited workforce information for policy decision‐making (WHO 2024). Nepal, therefore, does not simply illustrate “too few nurses.” It reveals a more analytically difficult configuration: numerical production alongside institutional scarcity, registration abundance alongside constrained authority, and professional expansion alongside fragile local voice. Importantly, I do not present Nepal as representative of all LMICs. Rather, I use it as a theory‐generating case for settings in which several conditions converge in varying combinations: rapid educational expansion, uneven labour‐market absorption, strong outward migration pressure, and weak institutional authority for nursing. Where those conditions are present, the concepts developed in this paper may travel; where they are absent, their applicability must be more cautiously delimited.

This paradox is visible well before nurses cross borders. In a mixed‐methods study of pre‐registration nursing students in Nepal, Poudel and colleagues found that 92.5% expressed some intention to migrate, with ‘further study abroad’ emerging as the leading rationale (Poudel et al. 2018). Previous research on undergraduate nursing students in Nepal has found that migration is driven not only by aspirations for professional development but also by institutional neglect, societal pressures, and perceptions that the nursing profession is “disposable” (Ghimire et al. 2024). Read together, these studies suggest that migration pressure is not simply a post‐qualification labour market event. It is cultivated during the professional development phase, emerging from the interplay between the expectations set forth by nursing education and the realities shaped by institutional dynamics.

This article, therefore, argues that the deeper crisis of nursing in Nepal and, by extension, in many LMICs, is a crisis of nursing sovereignty: the inability of nurses to shape the ends, authority, knowledge, and institutional future of their own profession. Sovereignty here does not mean separatism or nationalist closure. It names the minimum collective capacity required for nursing to function as more than exportable care labour: to define what counts as nursing knowledge, to participate authoritatively in governance, to secure material and educational resources for ethical practice, and to envision sustainable futures that can influence and guide the nursing profession's direction. The urgency of this argument is amplified by a global labour market in which one in seven nurses is foreign‐born, the proportion rises to 23% in high‐income countries, and only one quarter of low‐income countries report structured leadership development opportunities for nurses (WHO 2025b).

From this standpoint, the article makes three conceptual contributions. First, it develops the concept of epistemic drain to demonstrate that so‐called brain drain depletes not only the workforce but also mentorship, professional memory, pedagogical continuity, policy influence, and the local credibility of nursing as a form of knowledge discipline. Second, advances the claim that Nepali nursing is often professionalised for exit: educated in ways that enhance global mobility without proportionately increasing local voice, jurisdiction, or institutional authority. Third, it proposes nursing sovereignty as a normative framework for reimagining nursing in LMICs beyond retention rhetoric alone. The following argument does not assert that migration is inherently harmful or that workforce planning is unimportant; instead, it contends that both factors are inadequate unless we examine nursing as a profession whose authority can be systematically undermined, even as its labour heavily relies on it.

## 2 Philosophical Approach and Methodological Orientation

This article is a conceptual original contribution rather than an empirical report. Methodologically, it combines critical interpretive synthesis (CIS), philosophical analysis, and case‐guided theory building. Here, CIS is understood not as a protocol for exhaustive retrieval alone, but as a systematic yet iterative interpretive review aimed at engaging with diverse literatures. Its purpose is to facilitate explanatory critique, refine concepts, and develop synthetic constructs (Depraetere et al. 2021; Dixon‐Woods et al. 2006; Perlman et al. 2026). Operationally, the synthesis was guided by two iterative questions: *how should nursing in Nepal and comparable LMIC settings be understood when workforce expansion coexists with weak professional authority*, and *what conceptual resources best explain that pattern?* Electronic searching was conducted iteratively across PubMed/MEDLINE, CINAHL, Scopus, Web of Science, and Google Scholar, supplemented by backward and forward citation tracking and targeted searching of major policy sources. Search terms were combined and refined to focus on nursing, Nepal, LMICs, migration, brain drain, professional autonomy, epistemic injustice, decolonisation, care, education, mentorship, governance, and digital health. The included sources comprised peer‐reviewed journal articles, major policy reports, and foundational scholarly monographs that made substantive conceptual or empirical contributions to the paper's central problem. Sources were excluded when they focused exclusively on non‐nursing cadres, decolonisation, or migration and workforce issues with no meaningful bearing on authority, education, epistemic legitimacy, or institutional design. The aim was not statistical comprehensiveness, but interpretive adequacy: to assemble a body of literature capable of sustaining rigorous conceptual explanation rather than mere descriptive accumulation (Depraetere et al. 2021; Dixon‐Woods et al. 2006; Kuchenmüller et al. 2022). Because CIS depends on interpretive credibility rather than mechanical reproducibility, reflexivity was treated as integral to rigour. Literature was preserved not solely when it reinforced the developing argument; rather, studies and frameworks that introduced complexities—including research on remittances, skills transfer, health systems strengthening, and the agency of migrants—were intentionally integrated. This approach aimed to mitigate selection bias and rigorously assess the robustness of the conceptual assertions presented in the paper. The interpretive process involved a continuous comparison of Nepal‐specific studies, broader reviews of LMICs, and philosophical texts. Concepts were refined when strong convergences emerged and were qualified when the evidentiary base remained illustrative (Depraetere et al. 2021; Dixon‐Woods et al. 2006; Peddle 2022). Thus, the relationship between evidence and argument is staged rather than conflated: empirical and policy literatures highlight recurring patterns, while philosophical analysis explores their normative and epistemic significance.

With this methodological logic established, the article's first theoretical lens is social epistemology. In discussing testimonial injustice, I refer to the diminished credibility of a speaker due to identity‐based biases; by hermeneutical injustice, I refer to the absence or distortion of shared interpretive resources needed to make sense of a social experience. Hence, Fricker's account of testimonial and hermeneutical injustice provides a vocabulary for analysing how actors may be mistreated specifically in their capacity as knowers, while Code's feminist epistemology insists that knowledge is always situated and that epistemic responsibility is inseparable from social location (Code 1991, 2006; Fricker 2007). Dotson's concept of epistemic oppression extends this analysis by highlighting the persistent exclusion of subordinated groups from participation in knowledge production, while Medina's work on epistemic resistance helps illuminate how credibility deficits are collectively sustained and how they might be challenged (Dotson 2014; Medina 2013). These resources are especially useful for nursing because they allow the article to analyse not only the distribution of nurses but also that of credibility, interpretive authority, and institutional audibility.

A second lens is the feminist ethics of care and relational autonomy. Relational autonomy is used here to denote agency exercised within webs of dependence, recognition, and institutional constraint, rather than autonomy imagined as strictly individual independence. Sherwin (1992) feminist health ethics and McLeod and Sherwin's account of relational autonomy reject the notion that agency can be evaluated apart from structures of oppression, social dependence, and institutional constraint (McLeod and Sherwin 2000). Tronto's political theory of care goes further, showing that care is not merely an interpersonal virtue but also a question of how societies allocate responsibility, vulnerability, and power (Tronto 2013). Puig de la Bellacasa then expands care into an ethico‐political practice attentive to neglected labours, material entanglements, and the often ambivalent conditions under which caring work is organised (Puig de la Bellacasa 2017). Together, these approaches enable the article to treat autonomy not as an individual possession, but as an institutional accomplishment—or failure.

A third lens is a decolonial and political‐economic critique. Following Tuck and Yang, “decolonisation” is approached cautiously, precisely because its conceptual force is lost when it is reduced to a metaphor for generic reform (Tuck and Yang 2012). In nursing, Rosario and colleagues show that the term has already acquired multiple, sometimes competing meanings, while Iradukunda underscores how coloniality continues to shape what counts as valid knowledge in nursing discourse (Iradukunda 2023; Rosario et al. 2024). Here, decolonial thought functions less as a declarative label than as a critical test: whether a reform redistributes power, revalues marginalised knowledges, and alters the material conditions through which nursing is governed. Moreover, this article connects migration to the scholarship on global care chains and nursing migration studies, which examine ‘care mobility’ as a structured political economy rather than merely a collection of individual choices. Yeates characterises care transnationalisation as a socio‐spatial process, while Ortiga illustrates how nursing education in countries that send migrants actively cultivates labour for transnational markets (Ortiga and Rivero 2019; Yeates 2012). Additionally, the WHO Global Code of Practice offers a complementary normative framework for ethical recruitment and the protection of source countries (Brugha and Crowe 2015; WHO 2010).

The article is also informed by the sociology of professions, particularly Abbott's examination of jurisdictional struggles and Freidson's characterisation of professionalism as a unique method of organising expert work (Abbott 1988; Freidson 2013). These works elucidate how a profession can broaden its credentials without necessarily achieving equivalent authority. This issue is further exacerbated by spatial and technological arrangements. Dunn's recent analysis of nursing's “diminishment by design,” together with work on how space structures interprofessional collaboration, shows that visibility, proximity, and exclusion are materially organised rather than merely symbolic (Dunn 2025; Oandasan et al. 2009; Zook and Sailer 2022). Digital health and pedagogy are therefore treated here not as ancillary topics, but as a new terrain of inequality: The integration of generative artificial intelligence (gen AI) and advanced digital tools is progressively transforming nursing education and practice. However, the ongoing digital divide presents a significant challenge, potentially perpetuating disparities in educational and professional opportunities, even as it is framed as a driver of innovation (Bhoyar et al. 2024; El Arab et al. 2025; Makhene 2023).

Finally, methodologically, Nepal is not treated purely as a local example to which external theory is applied; rather, it is approached as a theory‐generative case. By this, I mean an analytically strategic case used to elaborate and refine concepts rather than to stand as a statistically representative sample of a larger population. Nepal is utilised instrumentally because it is a setting in which workforce expansion, outward migration, educational strain, weak policy voice, and uneven institutional recognition converge with unusual clarity. The objective is analytical generalisation—using one case to sharpen frameworks that may travel under comparable conditions (Ridder 2017; Ylikoski 2019). By reading Nepal through these philosophical and interdisciplinary lenses, the article seeks to generate concepts—epistemic drain, professionalised for exit, and nursing sovereignty—that are grounded in a specific reality yet analytically portable across other LMIC nursing contexts.

## 3 Nepal as a Theory‐Generating Case: Abundance, Scarcity, and the Paradox of Production

Nepal is analytically revealing not because it serves as a direct substitute for all LMICs, but because it concentrates several recurring tensions within lower‐resource health systems with unusual clarity. These include rapid educational expansion, uneven labour‐market absorption, rural–urban maldistribution, and strong outward mobility pressures. Consequently, Nepal is approached here as a strategically selected case for theoretical concentration rather than as a statistical representative. Its methodological value lies in making visible, within a single setting, structural contradictions that are often widely dispersed elsewhere. On aggregate indicators, the country can appear comparatively well supplied: the WHO human resources for health profile reports a density of medical doctors, nurses, and midwives of 51.0 per 10,000 population in 2023, exceeding the 44.5 per 10,000 benchmark associated with the Global Strategy on Human Resources for Health. Yet the same profile identifies rural retention, international migration, transformative education, and strategic direction and governance as ongoing priorities, while also noting that distribution data are not available in a form sufficient to determine where practitioners are actually located or how they are mobilised (WHO 2024). Nepal, therefore, exposes a central methodological problem in nursing policy: administrative sufficiency and lived reality are not the same phenomenon.

The tension becomes more evident when we examine the current registration figures in relation to labour market trends. More than 128,000 nursing and midwifery registrations reflect a strong training and credentialing system, highlighting the profession's prominence (NNC 2026). Yet the workforce research has long shown that Nepal's problem is not reducible to numerical shortage. Adhikari's now‐classic study of workforce management described a severe rural–urban maldistribution: hospitals in Kathmandu carrying an oversupply of newly qualified nurses while facilities outside the valley remained chronically understaffed (Adhikari 2014). What Nepal reveals is a paradox of both abundance and scarcity: abundance in registers, yet scarcity in service.

A second contradiction lies in the relation between educational production and system absorption. The WHO profile shows that in 2023, Nepal had 2660 BSc Nursing and 2120 Diploma of Nursing and Midwifery seats, providing clear evidence of ongoing production capacity (WHO 2024). But expansion is not neutral. Historical analyses of the sector argue that the growth in nursing education since the liberalisation of the 1990s has been accompanied by commercialisation, uneven quality control, weak demand–supply dynamics, and an increasing orientation toward external labour markets rather than domestic system needs (Adhikari 2014; Subedi 2014). Seen comparatively, this is not a uniquely Nepali anomaly: other LMICs have also reported the coexistence of health‐worker shortages with unemployment, underemployment, or weak deployment systems, suggesting that production and utilisation diverge sharply where governance and financing remain misaligned (Nwadiuko et al. 2025). In this sense, Nepal and other LMICs are not just producing nurses; they are producing them within an educational economy whose growth can outpace the state's capacity or willingness to absorb them into stable, geographically equitable, and professionally meaningful roles.

A third tension concerns the relationship between aspiration and authority. Nepalese nursing is not marked by an absence of professional ambition; rather, it is marked by institutional environments that often fail to convert aspiration into voice. In a national study of graduate nursing faculty, only 36.8% reported satisfaction with their current job, and the strongest predictors of satisfaction were not salary alone but involvement in departmental decision‐making and adequate access to reference materials, suggesting that authority and knowledge resources are central to professional viability (Sapkota et al. 2019). Likewise, a study of new nursing graduates in Nepal found that transition to practice was experienced through “getting hit by reality,” “losing confidence,” and “feeling unsupported,” with participants describing being ignored, accused, overburdened, and controlled by seniors in hierarchical workplaces (Gautam et al. 2023). This issue extends beyond the inherent challenges of work; it lies in how the prevailing institutional framework often denies the mechanisms of recognition necessary to transform professional ambition into sustained authority.

The fourth tension is between global mobility and local epistemic continuity. A study found that 92.5% of nursing students in Nepal expressed some intention to migrate (Poudel et al. 2018). That finding is often read as evidence of workforce exit; it is equally evidence of the social horizon within which nursing is imagined. Continuing professional development (CPD) data reinforce the same pattern. In a study of nursing stakeholders, Simkhada and colleagues identified barriers to post‐registration development at policy, organisational, and individual levels, including centralised training, lack of funding, lack of backfill staff, poor continuity of training, political influence, and the absence of mandatory CPD requirements for licence renewal (Simkhada et al. 2023). When professional development is sporadic, inconsistent, and poorly integrated into the institutional framework, employee mobility not only depletes the labour force but also disrupts the continuity of mentorship and the overall process of professional socialisation. The analytical significance of Nepal, then, does not rest on the simple assertion that all nursing systems in LMICs are identical. Rather, it lies in illustrating how both “oversupply” and “shortage” can coexist when four specific conditions are present: rapid educational expansion, uneven institutional absorption, significant outward migration pressure, and limited professional authority. It is within this context that insights derived from Nepal are most likely to be applicable to other LMIC environments.

## 4 From Brain Drain to Epistemic Drain

The language of *brain drain* has long been used to describe the emigration of skilled professionals from poorer to wealthier labour markets, and nursing migration research has often retained that grammar: exodus of skilled professionals, source countries lose labour, and destination countries gain urgently needed staff (Ghimire et al. 2025; Kingma 2001). That framing is important, but it is too narrow for the problem this article seeks to name. It captures movement more easily than consequence, and it captures headcounts more readily than institutional afterlives. In nursing, the issue extends beyond labour shortages. It also involves a profession's diminishing ability to sustain itself as a confident, authoritative, and locally grounded knowledge discipline. Accordingly, I use the term *epistemic drain* to denote a more specific, delimited problem than brain drain alone. In this article, the term does not refer to every adverse consequence of migration, nor does it imply that all downstream harms move together in a single, empirically uniform causal chain. Rather, it refers to the weakening of the profession's capacity to reproduce, circulate, and authorise its own knowledge when migration, attrition, and chronic institutional undervaluation deplete the personnel and structures that sustain that capacity.

Epistemic drain differs from workforce depletion in both object and scale. Labour loss is quantitative: fewer nurses available to staff wards, supervise students, manage workloads, or fill public posts. Epistemic drain is qualitative and institutional: the erosion of the social and organisational conditions that allow nursing knowledge to be taught, trusted, developed, and exercised with authority. It names the weakening of what might be called nursing's *epistemic infrastructure*, the mentors who model judgment, the educators who sustain standards, the senior clinicians who carry professional memory, the researchers who convert practice into evidence, and the policy actors who render nursing knowledge audible in governance. A health system can therefore continue to produce nurses numerically while progressively losing its ability to reproduce nursing epistemically. Put differently, it can maintain throughput while hollowing out authority. Nancy Fraser's language helps clarify what is at stake here: brain drain is often framed primarily as a problem of redistribution, but epistemic drain reveals that it is equally a crisis of recognition and representation—of whose knowledge counts, and who is present where decisions are made (Fraser 1998, 2009).

For analytic clarity, however, these concepts require distinct boundaries. *Brain drain* refers to the cross‐border loss of trained personnel. *Workforce depletion*, more broadly, refers to reduced staffing capacity, whether caused by migration, attrition, illness, or weak recruitment. *Care drain* foregrounds the transnational transfer of caring labour and its social reproductive consequences for sending countries (Kaelin 2011; Yeates 2012). *Epistemic injustice*, by contrast, concerns the negligence of persons in their capacity as knowers, such as credibility deficits or interpretive marginalisation (Fricker 2007). While *epistemic drain* intersects with each of these phenomena, it is not reducible to any single one; rather, it constitutes a profession‐level erosion of epistemic infrastructure. In the argument that follows, I apply this concept across three analytically distinct domains: (1) *transmission*, referring to mentorship and pedagogical continuity; (2) *production*, referring to research capacity and scholarly reproduction; and (3) *authorisation*, referring to policy voice and professional credibility.

One domain in which this erosion is especially visible is mentorship and pedagogical continuity. Mentorship literature from LMICs shows that the crisis is not only about staff numbers but also about the fragile transmission of ‘know‐how’ across generations of practice. A scoping review argues that LMIC human resources crises compromise not only practitioners’ availability but also workforce training and development, and identifies mentorship as an underdeveloped yet potentially powerful strategy for improving the quality of care (Schwerdtle et al. 2017). Hoover and colleagues similarly note that some of the largest in‐service nurse mentoring programmes in the world are located in LMICs, precisely because mentoring is being used to compensate for fragile support systems and uneven clinical preparation (Hoover et al. 2020). The ongoing educator shortages reveal the fragility of this situation. A study by Berland et al. (2020) identifies a shortage of qualified nurse educators as a significant barrier to the global nursing supply, particularly in LMICs. Additionally, Hookmani et al. (2021) elucidate the need to restructure mentorship programmes in Pakistan, as their findings indicate that prevailing supervisory practices fall short of fostering a supportive and professionally enriching environment. What is lost when experienced nurses depart is not just labour power, but also the continuity of their training and knowledge transfer.

A second dimension of epistemic drain is the attrition of research capacity and scholarly reproduction. Edwards et al. (2009) conducted a foundational review on nurse‐led research capacity in LMICs and identified several key barriers: a scarcity of senior mentors, limited graduate opportunities, disciplinary power hierarchies, and chronic underfunding of nursing research. These factors collectively hinder nurses’ ability to engage in and lead research initiatives. More recent studies indicate that these challenges remain, though they are now articulated with more sophisticated conceptual language. Buser et al. (2024) contend that strengthening research capacity in LMICs should be viewed as a sustainable process rather than a singular training event, as high‐quality research relies on institutional ecosystems that foster skill development, leadership, and continuity over time. The emergence of remote writing and virtual research‐capacity initiatives confirms both the seriousness of the gap and the ingenuity of attempts to address it. Programmes such as Writing to Improve Nursing Science (WINS) were created precisely because nurse scholars in LMICs often lack access to publication opportunities, mentorship, and doctorally prepared faculty (Sun et al. 2019). While recent reviews of virtual research‐capacity strengthening demonstrate that online tools can enhance confidence and skills, it still encounters barriers such as a lack of research culture and uneven infrastructure (McGuire et al. 2025). In essence, epistemic drain does not denote a lack of intelligence; rather, it indicates the decline of the fundamental elements that support intelligence, such as enduring scholarship, effective leadership, and the preservation of disciplinary knowledge.

A third dimension concerns policy voice and professional representation. If nurses are numerically central yet politically marginal, then migration does more than reduce staffing; it can diminish the very cohort most likely to convert frontline experience into policy influence. Rasheed et al. (2020) conducted an integrative review that identified persistent barriers to nurses’ involvement in political and policy matters. These barriers include limited knowledge, organisational constraints, and broader structural exclusions. A systematic review by Josephine Etowa et al. (2023) focusing on LMICs reinforced this observation, revealing that nurses and midwives continue to have minimal participation in policy development. Marginal representation emerged as a recurring theme, emphasising the need to reconstruct systemic power relations so that nurses can co‐lead policy rather than simply implementing it. Additionally, a recent review by Touzami et al. (2025) emphasises this issue, demonstrating that nurses, who are vital to primary health care, particularly in underserved and remote areas, remain under‐recognised due to structural, educational, cultural, and policy‐related barriers. Hence, ‘epistemic drain’ refers to a dual phenomenon: experienced nurses are leaving the profession, while those who stay are compelled to operate within healthcare institutions that undervalue their expertise.

This distinction between migrating and undervalued nurses matters because it changes what counts as harm. A redistribution frame asks how many nurses have gone and what it costs to train them. An epistemic frame examines what happens when a profession loses mentors more rapidly than it can replace them. In Nepal, recent work has begun to empirically reveal these dynamics, with nursing students describing the absence of role models and an “eroding ideal” of the profession, and nurse managers depicting the exodus of staff as a source of moral distress and organisational fragmentation (Ghimire and Neupane 2025, 2026b). These studies indicate that the most significant impact of migration may not be evident solely at the moment of departure, but rather in aspects that become increasingly difficult to restore afterwards. Seen in this light, epistemic drain is not a metaphorical embellishment of brain drain; it is a distinct analytical concept. It shifts focus away from mobility alone and towards the weakening of nursing's epistemic infrastructure: mentorship, pedagogy, memory, scholarship, representation, and credibility.

## 5 Professionalised for Exit: Migration as a Rational Moral Response

The migration of Nepali nurses is frequently framed in moralistic terms, portrayed as disloyalty, a lack of commitment, or an opportunistic escape. Such assessments are philosophically flawed because they fail to address the ethical issue at hand. The central question is not whether individual nurses exhibit sufficient patriotism, but rather whether the institutional conditions that compel them to remain are professionally and morally sustainable. Across LMICs, the evidence consistently indicates that health worker migration is driven more by systemic factors than by individual aspirations. Key drivers include inadequate compensation, job insecurity, limited opportunities for career advancement, and workplace violence, among other structural pressures. At the same time, a balanced account must acknowledge that migration is not universally narrated as loss. Nurse migration has also been associated with professional development, improved income for families, remittances, and, at least in principle, forms of skills circulation or diaspora contribution to source‐country systems (Ghimire and Neupane 2026a; Kingma 2001; Lorenzo et al. 2007). In Nepal specifically, diaspora and expatriate contributions to the health sector have been documented, but the same literature also shows that they are weakly institutionalised, poorly recorded, and unevenly integrated into workforce planning (Ghimire 2026b; Ghimire and Neupane 2026a). These possibilities, therefore, complicate, but do not dissolve, the structural ethics of exit.

This is the context in which the sub‐thesis of this article becomes clearer: in Nepal, nursing is often *professionalised for exit, not for voice*. The point is not that nursing education intentionally trains students to leave, nor that migration produces no benefits. It is that credentialing opens transnational mobility more reliably than it opens local jurisdiction, policy influence, or secure professional futures. In some settings, migration may even function as a labour‐market safety valve, absorbing domestic underemployment or oversupply in urban centres; Adhikari's qualitative study of workforce management in Nepal, for instance, described an oversupply of new nurses in Kathmandu alongside staffing deficits elsewhere (Adhikari 2014). Yet this does not count as a vindication of the system. It more plausibly indicates a failure to align educational production with equitable institutional absorption. Research conducted in Nepal exemplifies this pattern; in a mixed‐methods study, the intention to migrate among Nepali nursing students was significantly correlated with diminished professional identity and with nursing being a non‐preferred career choice (Poudel et al. 2018). More recent qualitative research indicates that migration ambitions are formed early and are influenced by a widening gap between policy rhetoric about strengthening nursing and the persistent realities of undervaluation, hierarchy, safety concerns, social pressures, and peer influence (Ghimire et al. 2024). What is being produced, then, is not simply a workforce with transferable skills, but a professional imagination in which departure appears more plausible than local influence.

A relational understanding of autonomy clarifies why this matters. As MacDonald (2002) argues, nurse autonomy is best understood not as independence from context but as meaningful self‐direction within relations of interdependence. Migration decisions are therefore never purely individual acts; they are mediated by family obligations, institutional constraints, social recognition, economic insecurity, and the range of futures a health system makes imaginable. De Haas's ‘aspirations–capabilities’ framework is especially useful here because it treats mobility and staying as complementary forms of migratory agency: the relevant freedom is not movement alone, but the capability to choose where to live, including the option to remain (de Haas 2021). Read alongside this, Carling and Schewel's reformulation of aspiration and ability further clarifies that migration agency cannot be reduced either to structural coercion or to voluntarist preference; movement occurs when aspiration and the practical ability to act align, while immobility may be either chosen or constrained (Carling and Schewel 2018). Applied to Nepali nursing, this yields a sharper ethical insight. When local systems fail to provide a reasonably secure income, credible postgraduate pathways, professional recognition, or meaningful influence, “staying” ceases to be a neutral baseline for the moral justification of migration; instead, it becomes a constrained choice.

At the same time, to say that exit is morally intelligible is not to romanticise it. The ethical burden falls in two directions. For those who remain, constrained agency can become morally corrosive. Nursing ethics scholarship has shown that moral distress arises not only from difficult clinical decisions but from broader social, political, and organisational constraints on moral agency (McCarthy and Monteverde 2018; Peter and Liaschenko 2013). In Nepal, nurse managers have described this terrain precisely: resource allocation dilemmas, staffing collapse, compromised care, and the cumulative ethical toll of trying to uphold standards within a system thinned by out‐migration (Ghimire and Neupane 2025). For those who leave, migration is no guaranteed deliverance. Longitudinal studies tracking Nepali nurses indicate that their aspirations to work abroad are often complicated by cultural dislocation, emotional strain, and unmet expectations regarding professional advancement and financial rewards. Nevertheless, some nurses manage to develop new forms of resilience, while others discover unexpected avenues to fulfilment within their home country (Ghimire and Qiu 2025). More broadly, critiques of health worker migration have challenged the optimistic assumption that remittances and training opportunities automatically compensate for source‐country loss; in some cases, the aggregate economic and service costs to source systems exceed these gains, however, the actual returns for long‐term system strengthening remain limited (Eaton et al. 2023). Previous critiques of Nepal‐UK nurse migration have highlighted a critical issue: the volatility of destination‐country recruitment frameworks can create conditions that effectively “trap” nurses. This situation constrains their choices, resulting in an environment that is neither entirely liberated nor adequately safeguarded (Adhikari and Grigulis 2014).

The ethical conclusion is therefore more demanding than either condemnation or celebration. The migration of nursing professionals from Nepal should be understood as a morally driven response shaped by structural factors related to an unstable future, rather than as a reflection of inadequate moral character or a straightforward success narrative. A just response does not begin by urging nurses to remain in their positions, nor should it presuppose that remittances, diaspora connections, or potential skills transfer adequately compensate for ethical considerations. It begins by asking what would make staying a viable, respected, and future‐bearing professional choice while also enabling mobility under conditions of fairness rather than extraction. Until the Nepali nursing profession is structured to not only facilitate credential recognition abroad but also to uphold its own authority, gain institutional respect, and ensure sustainable career opportunities domestically, the decision for nurses to seek employment opportunities overseas will continue to be both logical and justifiable.

## 6 Educational Erosion and the Making of Diminished Knowers

A workforce crisis not only depletes the capacity for service delivery but also fundamentally alters the contextual landscape in which professionals acquire knowledge and competencies. In nursing, educational erosion is therefore not a secondary effect of shortage or migration, but a constitutive mechanism of professional diminishment. The challenge does not stem from a lack of oversight or educational opportunities for students. Rather, it lies in their initiation into the profession, which is characterised by a continuous interplay of absence, improvisation, and muted authority. What is at stake is a form of epistemic apprenticeship: the process through which novices learn not only techniques, but what counts as credible knowledge, and how professional judgment is enacted in practice. Educational experiences for students extend beyond resource scarcity; rather, their identities and capabilities are fundamentally shaped by the limitations it imposes (Hunter and Cook 2018; Raso et al. 2019).

When workforce pressures intensify, the clinical setting ceases to operate as a space of guided professional formation and instead becomes a site of tactical survival. Recent research conducted in Nepal elucidates this transformation. Final‐year nursing students described compromised learning opportunities, isolation, lack of supervision, and an increasing need to seek answers outside the clinical relationship itself; in one of the study's most striking findings, students referred to gen AI as a form of “digital mentor” in settings where human mentorship had become too sparse or too hurried to rely on (Ghimire and Neupane 2024). Overburdened mentors do more than miss teaching opportunities; they change the developmental fabric of professional formation. Students begin to experience knowledge as fragmented, externally scavenged, and detached from the embodied judgment of trusted practitioners. The resulting lesson is subtle but consequential: nursing knowledge comes to appear less as a disciplined, collectively sustained practice of inquiry and more as something pieced together under pressure.

This damage to nursing knowledge is intensified by the hidden curriculum. Nursing education has long been recognised as a domain in which what students absorb exceeds what formal syllabi declare. Raso et al. (2019) define the hidden curriculum as the set of unintended, culturally acquired lessons through which students internalise professional values and identities, often without explicit acknowledgement by educators. Whereas Hunter and Cook (2018) show that new graduate nurses’ professional socialisation occurs through encounters with both desirable and undesirable role models; attitudes, decision‐making patterns, and behaviours observed in clinical practice become templates for future professional conduct. In that light, where nurses are too stretched to teach, too fatigued to explain, or too constrained to challenge unsafe or dismissive practices, students learn more than procedural shortcuts. They learn what must not be asked, which forms of knowledge are not worth voicing, and how to survive within hierarchy by withholding rather than articulating judgment.

The language of institutional betrayal helps clarify the moral structure of this experience. In a concept analysis, Brewer (2021) describes institutional betrayal in nursing as a deep violation of trust or moral standards committed by an institution against those who depend on it. While developed in relation to nurses more broadly, the concept is analytically apt for educational settings in which institutions promise clinical formation but deliver chronic absence, weak supervision, and the normalisation of neglect. Read alongside recent Nepali work showing that students experience an “eroding ideal” of nursing and a “ripple effect of absent role models,” institutional betrayal becomes a way of naming the deeper injury: the very institutions charged with forming nurses as ethical and competent professionals may instead socialise them into disillusionment (Ghimire and Neupane 2026b). The betrayal here is not dramatic abandonment but the quieter breach of educational trust.

A further dimension of educational erosion lies in weak **research socialisation**. Edwards and colleagues argued more than a decade ago that without adequate educational preparation and enabling environments, nurses in LMICs are unlikely to initiate research or demand research‐capacity opportunities (Edwards et al. 2009). That concern has not disappeared. Continuing professional development requirements in LMICs remain uneven, with participation often limited by funding, infrastructure, and access barriers, and with many settings lacking stable, mandated CPD structures altogether (Merry et al. 2023). When individuals enter a profession in which academic research is not well established, and ongoing education relies more on personal initiative than on structured pathways, they are conditioned to view nursing as a practice focused solely on implementation rather than inquiry.

Therefore, educational erosion is not just an unfortunate consequence of workforce instability; it is one of the earliest indicators of a diminishing nursing authority. The profession does not first experience a decline in policy discussions and only subsequently in educational settings and clinical environments. Rather, its erosion begins within the very ecosystem through which future nurses learn the essence of nursing. *Epistemic drain* begins here: in weakened mentorship, in hidden curricula of silence and compliance, in attenuated research cultures, and the replacement of systematic training with ad‐hoc improvisation. By the time students graduate, the erosion has already become part of what they understand professional life to be.

## 7 Care Without Sovereignty: Devaluation, Obedience, and Institutional Silence

If the previous sections have shown that Nepali nursing is shaped by migration pressure, educational erosion, and institutional fragility, the deeper philosophical diagnosis is that it often operates as **care** without sovereignty. By *care without sovereignty*, I mean a condition in which nursing labour is publicly required and morally valorised, yet nurses have limited authority to define the terms, value, and institutional organisation of that labour. This is an interpretive diagnosis rather than an empirically exhaustive label: the aim is not to claim that all dimensions of Nepali nursing can be reduced to one condition, but to show that several recurring patterns are intelligible when read together through three linked themes—devaluation, disciplined obedience, and institutional silence. In Woodly et al. (2021), care is recasted as a political and material practice rather than a private virtue; in Dunn (2025), nursing subordination is treated as an effect of institutional design rather than historical factors; in Jackson (2026), hierarchy is shown to persist through managerial visibility and audit cultures; and in Rosario et al. (2024), “decolonisation” is rescued from buzzword status by reconnecting it to power, materiality, and epistemic legitimacy. Taken together, these works do not empirically establish that Nepal is a singular case of “care without sovereignty”; rather, they provide a conceptual vocabulary for interpreting Nepali evidence in which nursing is publicly indispensable and morally burdened, yet recurrently denied proportionate authority over its own institutional and epistemic terms.

The devaluation is not only economic; it also encompasses cognitive and symbolic dimensions, and Nepali nurses have repeatedly articulated this in collective action. In a grounded theory study of health‐worker protests, the findings showed that nurses’ demonstrations were animated not only by demands for pay and working conditions, but also by demands for qualified leadership, policy implementation, and “equitable recognition of professional roles,” with nurses explicitly challenging discriminatory hierarchies and gendered perceptions of nursing as a subordinate profession (K.C et al. 2025). While this evidence does not independently establish a totalising condition of systemic diminishment, it reveals that nurses themselves conceptualise their injustice not only as material deprivation, but as *misrecognition*: a refusal to treat nursing knowledge, labour, and professional standing as decision‐worthy. This pattern is further reflected in hospital‐based research on professional identity. K.C et al. (2025) found that while nurses report a high sense of personal calling, their engagement with professional organisations remains notably weak, with fewer than half holding membership. Read cautiously, this suggests a systemic tendency to cultivate devotion to service while actively neglecting the development of collective professional authority.

A selective Foucauldian reading helps explain why this pattern is so durable. The point is not to redescribe Nepali nursing as nothing but domination, but to illuminate how obedience can become normalised as a professional virtue. Purvis (2023) shows, through a Foucauldian reading of nursing ethics, that professional identity can be shaped through discursive mechanisms that both elevate nursing morally and subordinate it epistemically. Jackson's (2026) analysis of surveillance and performance monitoring in nursing academia extends this insight: hierarchy is sustained not only by overt command, but through expectations of visibility, pace, tone, and compliance. Dunn (2025) similarly shows how architecture, governance, and credentialing arrange nursing as perpetually available labour rather than as a fully authoritative discipline. Broader health‐care evidence reinforces this interpretive move. A scoping review by Essex et al. (2023) shows that hierarchy shapes not only formal rank but also what becomes acceptable to say, by whom, and at what moment, with substantial personal costs for those in less powerful positions. In Nepal, this disciplinary pattern is visible not only in hierarchy but in exposure. A multicentre study found that 64.5% of nurses had experienced some form of workplace violence in the previous 6 months, most often verbal violence, with perpetrators commonly including patients’ relatives and hospital employees (Pandey et al. 2018). Workplace violence does not, on its own, establish “care without sovereignty.” It does, however, suggest a work order in which nurses are expected to absorb hostility, distress, and institutional failure without equivalent power to define the conditions under which care is delivered. In such settings, obedience can cease to be a freely embraced professional ethos and become a practical strategy of survival instead.

The third pattern is institutional silence. This denotes not personal reticence, but the structured inhibition of speech within organisational environments—especially where voice carries risk and influence remains unevenly distributed. Recent qualitative synthesis confirms that organisational silence among nurses is shaped by seniority, fear, acquiescence, disregard, and institutional cultures that render speaking unsafe or futile (Zou et al. 2025). Related work on hierarchy and safety voice demonstrates that nurses’ willingness to speak up is heavily dictated by power relations and psychological safety rather than by knowledge deficits alone (Essex et al. 2023; Lee et al. 2021). Furthermore, Rosario et al. (2024) demonstrate that epistemic dominance is reproduced when subordinated forms of knowledge are treated as derivative, local, or merely experiential rather than authoritative. In nursing, such dominance often manifests as inclusion without influence: nurses may be praised as essential or consulted as stakeholders, while remaining systematically excluded from the sites where priorities are set and policies are crafted. Evidence from Nepal suggests this risk is highly tangible; accounts of collective mobilisation indicate that demands for epistemic justice emerge precisely when formal institutional channels fail to recognise nurses’ practical experience as legitimate contributions to decision‐making (K.C et al. 2025). Crucially, these dynamic highlights a systemic tendency rather than an absolute condition: while not every nurse is rendered silent, the evidence strongly supports a reading in which silence is structurally enforced rather than individually chosen.

Care without sovereignty, then, should be understood as a bounded philosophical diagnosis of structural injustice. It names a condition in which nursing is trusted to care, endure, and absorb, but is not equally trusted to define, govern, or transform the institutions through which care is organised. The Nepali studies cited here do not, by themselves, prove a complete theory of nursing subordination. They do, however, converge with broader scholarship in nursing philosophy in ways that make such an interpretation plausible. If sovereignty is the collective capacity to shape the ends and terms of one's own professional life, then what Nepal reveals is not a deficit of caring commitment, but a recurrent pattern in which care is extracted under conditions of constrained authority. That, I suggest, is one philosophical core of the present crisis.

## 8 Mobility Justice, Decolonisation, and the Ethics of Recruitment

Transitioning from the framework of migration ethics to that of mobility justice necessitates a shift in the normative inquiries we pursue. The focus should not be on the permissibility of nurses crossing borders; rather, we must challenge and reject the idea that professionals from source countries are duty‐bound to remain within their national boundaries against their will. The critical question, therefore, is whether cross‐border mobility is structured through just relations. The WHO Global Code of Practice on the International Recruitment of Health Personnel provides the clearest global starting point. Since its adoption in 2010, the Code has established ethical recruitment as a key component of efforts to enhance health systems, particularly in developing countries. It continues to serve as the primary global framework for balancing the rights, obligations, and expectations of source countries, destination countries, and migrant health workers (WHO 2010, 2025a). At the same time, some of the strongest defences of managed migration argue that bilateral agreements and regulated recruitment can, in principle, create safer mobility pathways, support remittances, enable skills circulation, and generate benefits for both sending and receiving systems when embedded in broader cooperation arrangements (Lorenzo et al. 2007; Squires and Amico 2014). The arguments presented warrant in‐depth examination as they highlight tangible benefits that a strictly loss‐centric perspective may overlook. Migration has the potential to enhance professional opportunities for individual nurses, promote diversity within destination healthcare systems, and foster the development of resources or transnational networks. Under certain circumstances, these dynamics can yield positive outcomes for households and institutions in the source countries (Lorenzo et al. 2007; Stokes and Iskander 2021). While this framework marks the beginning of mobility justice, it cannot encompass the entirety of the issue.

The concept of “ethical recruitment” can receive formal endorsement while lacking substantial depth in practice. Initial assessments of the WHO Code revealed widespread symbolic adoption, yet implementation has been inconsistent, with significant variations in national reporting and policy translation across countries (Brugha and Crowe 2015; Siyam et al. 2013). Indeed, the recent WHO report credits the ‘Code’ with increasing awareness, improving workforce data, and expanding the reporting of bilateral agreements, while also acknowledging a persistent gap between formal policy uptake and the actual delivery of mutual benefits to health systems (WHO 2025a). However, recent critiques have intensified, as Hanrieder and Janauschek (2025) contend that even those nations typically viewed as exemplary in their recruitment practices can repurpose ethical considerations into a legitimising discourse for ongoing extraction. They refer to this phenomenon as “liberal health worker extractivism”. Walton‐Roberts (2022) similarly shows that destination countries may invoke the Code while continuing to benefit from training investments made elsewhere, unless they also strengthen domestic workforce self‐sufficiency and support fair recognition and integration for incoming workers. Related health‐system reform scholarship further sharpens this point: ethical recruitment cannot substitute for robust domestic planning, training, and retention in destination countries, nor can it be treated as sufficient if source‐country systems remain structurally depleted (Aluttis et al. 2014; Walton‐Roberts and Bourgeault 2024). The ethical problem, then, is not migration itself, but mobility without reciprocity: destination‐country gain without source‐country investment, and circulation without structural redress.

This is where decolonisation becomes analytically useful— not as a prohibition on bilateralism, but as a practical test of whether a recruitment arrangement redistributes power or merely manages asymmetry more politely. A decolonial test asks: Does the arrangement strengthen local nursing education, retention, and leadership in the source country? Does it recognise the source country as a co‐equal partner in setting terms? Does it protect migrant nurses’ dignity after arrival, including recognition of prior experience, safe integration, and non‐exploitative career pathways? Or does it leave the deeper hierarchy intact, with richer systems drawing from poorer ones under the language of partnership? Eckenwiler's account of transnational justice is especially useful here because it moves beyond blame toward structural responsibility, asking how receiving systems are implicated in the injustices that make mobility unevenly costly (Eckenwiler 2014; Eckenwiler 2009). A decolonial reading, therefore, does not dismiss the possibility that bilateral agreements may be ethically preferable to fragmented, broker‐led recruitment. Rather, it asks whether those agreements alter the distribution of benefits, authority, and risk in practice.

The Nepal–UK Memorandum of Understanding (MoU) provides a concrete test case. Publicly, the agreement can be framed as orderly, bilateral, and therefore more ethical than unregulated recruitment. A nuanced interpretation would suggest that these structures may mitigate informal brokerage, enhance the transparency of mobility pathways, and establish more clearly defined trajectories for professional advancement than unregulated recruitment channels. Yet policy analysis has argued that it reproduces neocolonial dynamics if it principally serves the UK's staffing crisis without corresponding investment in Nepal's nursing system (Ghimire et al. 2024). Subsequent qualitative work with Nepali nurses deepens that critique: participants described the MoU as simultaneously promising and troubling, naming opportunities for individuals but also fears of domestic depletion, unequal bargaining power, and uncertainty about whether the arrangement represented genuine partnership or a more polished mechanism of extraction (Ghimire et al. 2025). Importantly, the available literature does not yet show that the Nepal–UK arrangement has generated robust source‐country co‐investment, credible return pathways, or durable system‐strengthening effects; rather, it shows that these are precisely the domains in which reciprocity remains contested or insufficiently specified (Ghimire et al. 2024; Ghimire et al. 2025). Furthermore, ethical obligations extend beyond national borders. Research on internationally recruited nurses in England indicates that, despite arriving with high levels of education and experience, migrants often face inconsistent support, role downgrading, inadequate speciality matching, and integration challenges. This emphasises a longstanding concern that even in destination countries, the safeguarding of professional dignity remains insufficiently addressed (Pressley et al. 2023).

Mobility justice, therefore, requires more than bilateral paperwork and “do no harm” rhetoric. It requires destination countries to work towards greater domestic self‐sufficiency, to invest in source‐country workforce development where they benefit from outward mobility, to ensure fair recognition and integration after migration, and to treat source countries not merely as labour reservoirs but as equal partners in shaping terms of exchange. A more candid assessment of the complex moral landscape of migration is essential: while remittances, skill transfer, and professional opportunities are indeed significant, they alone do not justify recruitment. True justice hinges on whether mobility takes place under conditions of reciprocity, institutional dignity, and the strengthening of systems at both ends of the migration corridor.

## 9 Toward Nursing Sovereignty: A Six‐Domain Normative Framework

**Figure 1:**
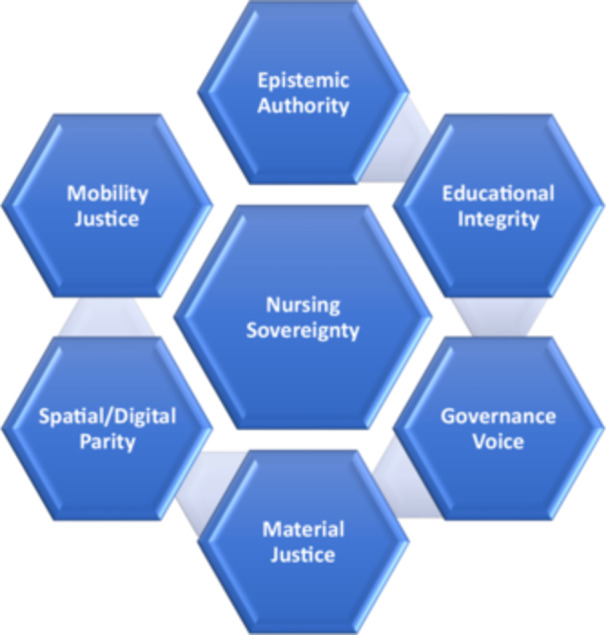
Six‐domain normative framework for nursing sovereignty.

If the preceding sections have served a diagnostic purpose, the current focus shifts to a reconstructive one. Nursing sovereignty should not be mistaken for professional isolation; rather, it denotes the essential institutional conditions required for nursing to function as a knowledge‐driven, ethically accountable, and politically impactful profession. This concept is best understood as a collective good, reflecting the capacity of nurses, as a unified profession, to influence how care is defined, taught, governed, and valued. Thus, *sovereignty* should be conceptualised as a framework for institutional redesign, rather than a motivational ideal. Fricker's account of epistemic injustice clarifies why credibility is central to justice, while Fraser's triadic model of redistribution, recognition, and representation clarifies why no single reform lever is sufficient (Fraser 1998, 2009; Fricker 2007). In this light, sovereignty moves beyond established paradigms. Whereas *professional autonomy* typically denotes discretionary authority within clinical practice, and *empowerment* describes access to resources and participation within existing hierarchies, *nursing sovereignty* operates as a comprehensive, cross‐level mandate (Gottlieb et al. 2021; Pursio et al. 2021). It concerns the profession's collective capacity to shape the epistemic, educational, governance, material, spatial‐digital, and transnational conditions of its own reproduction. Its originality lies not in discarding autonomy or empowerment, but in integrating them into a justice‐oriented model linking recognition, redistribution, and representation across domestic and transnational domains. In practical terms, the six domains below constitute a staged framework for institutional redesign, offering scalable entry points depending on system capacity, political conditions, and resource constraints (Figure 1).

The first domain is epistemic authority. Nursing sovereignty begins with the profession being recognised as a legitimate source of knowledge rather than as a downstream executor of other people's expertise. This means institutional recognition of situated, relational, and practical nursing knowledge in evidence production, quality improvement, and clinical decision‐making. Dunn's analysis shows that nursing subordination persists where institutions systematically discount nurses’ epistemic contributions. Moreover, the nurse prescribing literature demonstrates that jurisdictional authority can be deliberately codified when systems choose to do so (Dunn 2025; Kroezen et al. 2012). At the level of application, this domain points to concrete levers such as scope‐of‐practice reform, nurse‐led quality and safety committees, formal inclusion in evidence‐governance structures, and legal recognition of nursing judgment in defined areas of care. Nursing sovereignty, therefore, requires more than “valuing nurses”; it requires legal, organisational, and cultural arrangements in which nurses are authorised to interpret, decide, and lead within their scope without their knowledge being treated as secondary.

The second domain is educational integrity. A sovereign profession cannot be reproduced through pedagogical improvisation alone. Educational integrity requires mentoring capacity, curricular depth, continuing professional development, and protected pathways through which nurses are formed as practitioners, teachers, and researchers. Global nursing evidence now underscores the importance of education capacity, remuneration, leadership, and service‐delivery readiness as interconnected priorities. Research‐capacity scholarship likewise demonstrates that without sustained educational investment and enabling environments, nursing struggles to reproduce its own scholarly leadership (Edwards et al. 2009; Giordano et al. 2024; Merry et al. 2023; WHO 2025d). In practice, this domain identifies specific institutional priorities: funded educator positions, structured preceptorship and mentorship models, protected CPD budgets, clinical‐academic pathways, and research supervision capacity that is not left to informal goodwill alone. Hence, educational integrity entails cultivating an educational ecosystem in which nursing knowledge is both affirmed and actively encouraged. This holistic approach underscores the importance of fostering an environment that acknowledges the complexities of nursing education while promoting and supporting the development of nursing practice.

The third domain is governance voice. Sovereignty requires formal decision‐making power in the places where institutional priorities are set: ministries, regulatory councils, hospitals, universities, boards, and system‐design forums. Reviews of nurses’ policy participation continue to show that nurses are too often positioned as implementers rather than policymakers, while board‐governance scholarship demonstrates that nursing remains underrepresented in strategic decision‐making (Rasheed et al. 2020; Sundean and Gatiba 2022). Evidence on professional governance interventions further suggests that shared governance and empowered decision‐making structures can strengthen professional practice environments when institutionally supported (Kanninen et al. 2021). The actionable implication is that nursing representation must move beyond consultation to formal voting authority, protected board and committee seats, and clear decision rights in ministries, universities, and service‐delivery organisations. The governance voice thus serves as a critical representational framework that enables nursing to actively engage in the design and reform of health systems rather than reacting to their outcomes.

The fourth domain is material justice. Wages, workload, safety, and secure conditions are the preconditions of moral agency. The global nursing report identifies persistent inequities in wages and working conditions as central threats to sustainability (WHO, 2025 d). Work‐environment research has long shown that poor staffing and unsafe organisational environments are associated with burnout, job dissatisfaction, lower‐quality care, and suboptimal patient outcomes (Aiken et al. 2011; Li et al. 2024). Consequently, this domain necessitates actionable structural imperatives, including safe staffing mandates, robust violence‐prevention frameworks, equitable remuneration, and secure employment contracts. Ultimately, it demands the creation of material environments that render ethical practice structurally feasible, rather than relying upon the unsustainable heroic endurance of individual practitioners. A sovereign nursing profession, therefore, cannot be built on sacrificial labour alone. Material justice means that dignified work, manageable workload, and physical and psychological safety must be treated as constitutive of professional agency rather than as optional “retention incentives”.

The fifth domain is spatial and digital parity. Nursing sovereignty requires a presence in the physical and digital architectures in which authority is organised. Dunn's work and spatial studies of hospital design show that institutions encode hierarchy materially, while research on hospital architecture has argued that nurses should be understood as knowledge workers whose movement, visibility, and access are shaped by design (Dunn 2025; Zook and Sailer 2022). In the digital domain, the same principle holds: if gen AI tools, data systems, and learning platforms are designed without nursing input—or are unequally accessible—then epistemic exclusion is reproduced under a new technological guise (Ghimire 2026a). As a point of application, this domain requires nursing involvement in infrastructure planning, procurement, information governance, data standardisation, and generative AI design and oversight, so that nurses are not merely end‐users of systems built by others. Spatial and digital parity, therefore, means redesigning environments so that nurses are not peripheral users of systems built by others, but co‐constitutive actors in their formation.

The sixth domain is mobility justice. A sovereign profession must be able to circulate without being structurally drained. This means that ethical migration cannot be reduced to non‐coercive individual mobility or the existence of bilateral agreements. The 2010 WHO Global Code and its 2025 review make clear that migration governance must be tied to health‐system strengthening (WHO 2010, 2025a). Contemporary critiques of “ethical recruitment” show that destination countries can comply procedurally while continuing to extract developmental value from poorer systems (Hanrieder and Janauschek 2025; Walton‐Roberts 2022). In actionable terms, mobility justice demands structural reciprocity: source‐country co‐investment, transparent credential recognition, ethical employment protections, and supported pathways for circular migration. Migration governance must act as a lever for system strengthening rather than a substitute for it, serving as an integral component of equitable exchange rather than an obligation of repayment.

This framework is normative, yet deliberately pragmatic. Its implementation will inevitably be constrained by financial scarcity, educator shortages, fragmented regulation, deficient data systems, restrictive organisational cultures, and resistance from entrenched professional hierarchies. Empirical evidence on nursing policy participation demonstrates that time constraints, limited policy literacy, organisational exclusion, and unsupportive political environments persist as significant barriers to structural influence (Hajizadeh et al. 2021; Han and Kim 2024). Consequently, the framework is best operationalised as a phased progression rather than a simultaneous, comprehensive overhaul. In lower‐capacity settings, material justice and educational integrity must serve as first‐order stabilising priorities; once foundational stability is achieved, governance voice and epistemic authority can be institutionalised through board representation, scope‐of‐practice reforms, and nurse‐led quality structures. Subsequently, spatial‐digital parity and mobility justice can be embedded via procurement standards, infrastructure investments, and reciprocal international agreements. The framework is therefore aspirational in direction but actionable in architecture: it identifies the domains, institutional levers, and plausible sequences through which health systems can transition from symbolic inclusion toward genuinely shared authority.

Taken together, these six domains provide a portable framework for institutional redesign. Their philosophical strength is that they align closely with Fraser's triad: *recognition* is advanced through epistemic authority and spatial/digital parity; *redistribution* through material justice and educational integrity; and *representation* through governance voice and mobility justice. This alignment is also what distinguishes nursing sovereignty from adjacent autonomy and empowerment frameworks: it treats nursing not only as a profession seeking more discretion within existing systems, but as a collective actor requiring redesigned relations of *recognition, redistribution, and representation*. In this reading, nursing sovereignty is a justice‐oriented design principle for rebuilding health systems so that nursing can exist not as indispensable labour subordinated to others’ terms, but as a fully participating profession capable of shaping care, knowledge, and institutional futures.

## 10 Conclusion: Nepal and the Future of Nursing Philosophy

Nepal provides a distinct perspective on the challenges that nursing philosophy must address in the context of LMICs: the deepest crisis of nursing is not shortage alone, but the institutional production of nurses as essential yet disposable, mobile yet institutionally voiceless, and professionalised yet epistemically diminished. What this article has shown, however, is that these conditions are not best understood as a simple failure of retention, nor as evidence of inadequate professional commitment. They are symptoms of a more fundamental problem: a profession on which health systems depend, yet whose authority to define care, shape knowledge, influence institutions, and reproduce itself on just terms remains structurally constrained.

Seen in this light, Nepal is not a peripheral example of global inequity but rather a theory‐generating case whose significance lies in the convergence of rapid educational expansion, uneven institutional absorption, outward mobility, and constrained professional authority. Where these conditions coexist, the Nepali case reveals something of wider importance: a health system may expand training, celebrate nurses as indispensable, and even benefit from their global circulation while still failing to secure the conditions under which nursing can act as a knowledge discipline, a moral practice, and a political force.

Ultimately, the value of nursing philosophy lies in its capacity to drive institutional redesign. By advancing the six‐domain framework, this article demonstrates that the future of the profession in LMICs requires moving beyond the narrow policy focus of workforce retention. Hence, the imperative is clear: health systems must transcend efforts to simply retain staff, and instead cultivate the structural conditions required for nursing to exercise genuine sovereignty over its care, knowledge, and professional trajectory.

## Author Contributions

Conceptualization: Animesh Ghimire. Formal Analysis: Animesh Ghimire. Investigation: Animesh Ghimire. Writing – Original Draft Preparation and Writing – Review and Editing: Animesh Ghimire. The author approved the publication of the final version.

## Funding

The author has nothing to report.

## Ethics Statement

This study did not require ethical board approval because it did not contain human or animal trials.

## Conflicts of Interest

The author declares no conflicts of interest.

## Data Availability

Data sharing not applicable to this article as no datasets were generated or analysed during the current study.
